# Different Virus-Derived siRNAs Profiles between Leaves and Fruits in Cucumber Green Mottle Mosaic Virus-Infected *Lagenaria siceraria* Plants

**DOI:** 10.3389/fmicb.2016.01797

**Published:** 2016-11-09

**Authors:** Junmin Li, Hongying Zheng, Chenhua Zhang, Kelei Han, Shu Wang, Jiejun Peng, Yuwen Lu, Jinping Zhao, Pei Xu, Xiaohua Wu, Guojing Li, Jianping Chen, Fei Yan

**Affiliations:** ^1^State Key Laboratory Breeding Base for Sustainable Control of Pest and Disease, Zhejiang Academy of Agricultural SciencesHangzhou, China; ^2^Key Laboratory of Biotechnology in Plant Protection of MOA of China and Zhejiang Province, Institute of Virology and Biotechnology, Zhejiang Academy of Agricultural SciencesHangzhou, China; ^3^College of Life Sciences, Fujian Agriculture and Forestry UniversityFuzhou, China; ^4^Institute of Vegetable, Zhejiang Academy of Agricultural SciencesHangzhou, China

**Keywords:** virus-derived small RNAs, CGMMV, tissue-specific, NGS data analysis, *Lagenaria siceraria*

## Abstract

RNA silencing is an evolutionarily conserved antiviral mechanism, through which virus-derived small interfering RNAs (vsiRNAs) playing roles in host antiviral defense are produced in virus-infected plant. Deep sequencing technology has revolutionized the study on the interaction between virus and plant host through the analysis of vsiRNAs profile. However, comparison of vsiRNA profiles in different tissues from a same host plant has been rarely reported. In this study, the profiles of vsiRNAs from leaves and fruits of *Lagenaria siceraria* plants infected with Cucumber green mottle mosaic virus (CGMMV) were comprehensively characterized and compared. Many more vsiRNAs were present in infected leaves than in fruits. vsiRNAs from both leaves and fruits were mostly 21- and 22-nt in size as previously described in other virus-infected plants. Interestingly, vsiRNAs were predominantly produced from the viral positive strand RNAs in infected leaves, whereas in infected fruits they were derived equally from the positive and negative strands. Many leaf-specific positive vsiRNAs with lengths of 21-nt (2058) or 22-nt (3996) were identified but only six (21-nt) and one (22-nt) positive vsiRNAs were found to be specific to fruits. vsiRNAs hotspots were only present in the 5′-terminal and 3′-terminal of viral positive strand in fruits, while multiple hotspots were identified in leaves. Differences in GC content and 5′-terminal nucleotide of vsiRNAs were also observed in the two organs. To our knowledge, this provides the first high-resolution comparison of vsiRNA profiles between different tissues of the same host plant.

## Introduction

RNA silencing is a natural antiviral mechanism in plants and other eukaryotic organisms. Through the process, virus-infected plants produce virus-derived small interfering RNAs (vsiRNAs) which play important roles in host antiviral defense (Zhu et al., 2011; Szittya and Burgyán, 2013; Zhang et al., 2015). The endoribonuclease activity of the dicer-like proteins (DCLs) 2 and 4 is essential for the production of these vsiRNAs (Deleris et al., 2006). DCL4 mainly targets virus RNA to produce 21 nucleotide (nt) vsiRNAs, while DCL2 is responsible for the processing of 22-nt vsiRNAs when DCL4 is absent or its activity is inhibited (Xie et al., 2004; Deleris et al., 2006). Both vsiRNAs can guide the RNA-induced silencing complex (RISC) to slice viral RNA in a sequence-specific manner. In addition, two other plant DCLs, DCL1 and DCL3, are essential for the production of small RNAs (Henderson et al., 2006). DCL1 is mainly responsible for excising the stem-loop structures of primary microRNAs (miRNAs) into mature approximately 21-nt miRNAs that play key roles in post-transcriptional gene silencing (Blevins et al., 2006; Dong et al., 2008). The functions of these DCLs are overlapping and can be complemented. Very low levels of 21-nt vsiRNAs were produced by DCL1 in *dcl2*/*dcl3*/*dcl4* triple mutant plants infected with Cucumber mosaic virus (CMV), while 24-nt vsiRNAs were produced by DCL3 in *dcl2*/*dcl4* double mutant plants, indicating a compensatory role for these DCLs (Bouché et al., 2006; Deleris et al., 2006).

The biogenesis of vsiRNAs has attracted much attention over the past decade, but is still not comprehensively understood. Early studies indicated that vsiRNAs are mostly produced from double stranded viral RNA (dsRNA) replicative intermediates (RIs) in a process that generates almost equal numbers of vsiRNAs from the positive and negative strands (Ahlquist, 2002). In addition, the highly structured regions in a single stranded viral RNA (ssRNA) can also contribute to the biogenesis of vsiRNAs, resulting in many more vsiRNAs derived from positive strand rather than negative strand (Molnár et al., 2005; Szittya et al., 2010; Wang et al., 2010).

Next Generation Sequencing (NGS) technology has recently been used to investigate the vsiRNA profiles of various combinations of viruses and plants. In general, 21-nt vsiRNAs usually predominate in the population, there is a strong A/U bias at the first nucleotide of vsiRNAs, and vsiRNA-producing hotspots can be identified within the viral genome (Miozzi et al., 2013; Visser et al., 2014; Xia et al., 2014; Yang et al., 2014; Kutnjak et al., 2015; Li et al., 2016). Previous studies indicated that vsiRNAs are predominantly responsible for RNA silencing-mediated antiviral immunity and the main function of vsiRNAs is to target and degrade viral mRNA through post-transcriptional gene silencing in plants (Zhu et al., 2011; Zhang et al., 2015). Moreover, recent studies have shown that vsiRNAs may also occasionally regulate host mRNAs with near perfect complementarity. The first report of this phenomenon was the targeting of the chlorophyll biosynthetic gene (CHLI) of *Nicotiana* by siRNAs derived from CMV Y-satellite, resulting in the yellowing of the plant (Shimura et al., 2011; Smith et al., 2011). It has also recently been shown that the eukaryotic translation initiation factor 4A (eIF4A) of *Nicotiana benthamiana* can be targeted by siRNA derived from Rice stripe virus (RSV), resulting in leaf-twisting and stunting (Shi et al., 2016). These results indicate the complicated function of vsiRNAs during virus-host interaction.

Cucumber green mottle mosaic virus (CGMMV) is a member of the genus *Tobamovirus*, family *Virgaviridae*, and causes a serious disease of cucurbit crops with significant economic losses in several countries including Israel, China, Korea and Russia (Antignus et al., 1990; Ugaki et al., 1991; Kim et al., 2003; Slavokhotova et al., 2007; Liu et al., 2009). Recently, it was reported on melon in the United States (Tian et al., 2014). CGMMV can be transmitted mechanically on seeds and pollen, causing typical mosaic and mottling symptoms on leaves, as well as fruit distortion (Mink, 1993). Similar to other tobamoviruses, CGMMV is a single-stranded positive RNA virus with a 3′ tRNA-like structure, encoding four polypeptides including a 124- to 132-kDa protein, a 181- to 189-kDa read-through protein, a 28- to 31-kDa movement protein (MP) and a 17- to 18-kDa coat protein (CP) (King et al., 2011). The profile of CGMMV-derived siRNAs in infected leaves of cucumber was reported recently (Li et al., 2016). The present study reports markedly different vsiRNA profiles (abundance, polarity and hotspot distribution) between infected fruits and leaves of *Lagenaria siceraria*.

## Materials and methods

### Sample collection and total RNA extraction

Seeds of bottle gourd (*Lagenaria siceraria*, accession “Hangzhou gourd”) were sown in soil rich in organic matters in a greenhouse with the ambient temperatures between 20 and 25°C, and watered every 3 days to maintain ample soil moisture. At the two and a half leaf stage plants were mechanically inoculated with CGMMV virions on the two expanding leaves using sap from a previously infected plant. Approximately 100 mg of tissue was homogenized in 20 volumes of inoculation buffer (0.1M phosphate buffer, pH7.5, 0.2% sodium sulfite and 0.01M 2-mercaptoethanol), while the mock plants were only inoculated with inoculation buffer.

Three replicate samples of fruit and leaves from plants with typical CGMMV symptoms and from mock controls were collected for RNA extraction. Total RNAs were extracted from each sample using Trizol (Invitrogen, USA) according to the manufacturer's instructions. The presence of CGMMV infection in the tissues was confirmed with a One Step RT-PCR Kit (TOYOBO, Japan) following the product's protocol and using CGMMV specific primers (CG-F: 5′-GCTTACAATCCGATCACAC-3′; CG-R: 5′-ATTATCTATCTCAGCCCTAG-3′). The RNA quantity and quality from each sample was evaluated by denaturing agrose gel electrophoresis and a 2100 Bioanalyzer (Agilent, USA).

### Small RNA sequencing and raw data pre-processing

Approximately 5 μg of total RNA was extracted for the preparation of a small RNA library according to the protocol of TruSeq Small RNA Sample Prep Kits (Illumina, USA). Briefly, total RNA was resolved using denatured 8% polyacrylamide gel electrophoresis (PAGE) and small RNA fragments were isolated. After ligation of the 5′ and 3′ adaptors, the short RNA fragments were reverse transcribed using SuperScript II Reverse Transcriptase (Life Technologies, USA) and amplified by PCR. Finally, single-end sequencing (36 bp) was performed on an Illumina Hiseq2500 at LC-BIO (Hangzhou, China) following the protocol of the manufacturer.

After parsing small RNA sequences from the 3′ adaptor sequence, low quality and junk sequences, including transfer RNAs (tRNAs), ribosomal RNA (rRNAs), small nucleolar RNAs (snoRNAs), small nuclear RNAs (snRNAs), and repetitive sequences, were removed using the FASTX-Toolkit (http://hannonlab.cshl.edu/fastx_toolkit/). The remaining sRNA reads were collapsed to uniread sets and the reads of > 30-nt or <18-nt were discarded. Clean sRNA reads were used for further bioinformatics analysis.

### Bioinformatics analysis of sequencing data

To identify CGMMV-derived siRNAs, processed reads from each of the 12 *L. siceraria* libraries were mapped to the CGMMV reference genome (NCBI Accession No: KP868654) using Bowtie software (http://bowtie-bio.sourceforge.net) with one mismatch. To facilitate comparisons across different libraries, vsiRNA read numbers were normalized to “Reads Per Million” (RPM) based on the total small RNA read numbers of the corresponding library. All of the downstream analyses were performed using custom perl scripts and linux (Cent OS 6.5) bash script. For statistical analysis of the three biological replicates, one-way ANOVA analysis using Originpro 8.5 software was performed and values of *p* < 0.01 were considered significant. To avoid the inaccuracy of low copy sequences, sequences with <10 raw reads in each of the three replicates were removed (for the analysis of leaves or fruits specific vsiRNAs). Specific (Unique) vsiRNAs were extracted from the three replicates of each sample during this analysis. RNA secondary structures were predicted using RNAfold (http://rna.tbi.univie.ac.at/cgi-bin/RNAfold.cgi) with default parameters.

### Northern blot

Total RNA was isolated from plants with Trizol (Invitrogen, USA) according to the manufacturer's instructions. For northern blot of CGMMV RNAs, a DNA probe targeting CGMMV CP was synthesized with primers (5′-GCTTACAATCCGATCACAC-3′ and 5′-ATTATCTATCTCAGCCCTAG-3′) and labeled with DIG according to the manufacturer's protocol (DIG Oligonucleotide 3′-end labeling Kit, Roche, USA). For northern blot of positive-stranded CGMMV RNAs in leaves, a sequence (5′-CAACACAGGACCGTTGAGGAAAGCGTAAAAACCCGCACCTGGGAATCTAGAATTAATATCTACGACAGACGAGGGTAACGCA-3′) was synthesized and labeled as DNA probe, and its complementary sequence was used for detecting negative-stranded CGMMV RNAs. Another sequence (5′-CATAGCTCTGAGCTTTAACTACACTAAAGTCAGTTATAGATAAATACTTAAGAATGGAAAAATAGTTAGGGAGCAACTTATC-3′) was used for detecting positive-stranded CGMMV RNAs in fruits, and its complementary sequence was used for detecting negative -stranded CGMMV RNAs in fruits. Pre-hybridization, hybridization and signal detection were done according to the protocol of the DIG High Prime DNA Labeling and Detection Starter Kit II (Roche, USA).

### Tissue immunoblot

Tissue immunoblot was carried out as described previously (Andika et al., 2005). Primary anti-CP (1: 5000) polyclonal serum and secondary polyclonal AP-conjugated goat anti-rabbit IgG (1: 10 000) (Sigma, USA) were used for blotting according to the methods described before (Peng et al., 2011).

## Results and discussion

### Overview of small RNA deep sequencing data

**Figure 1:**
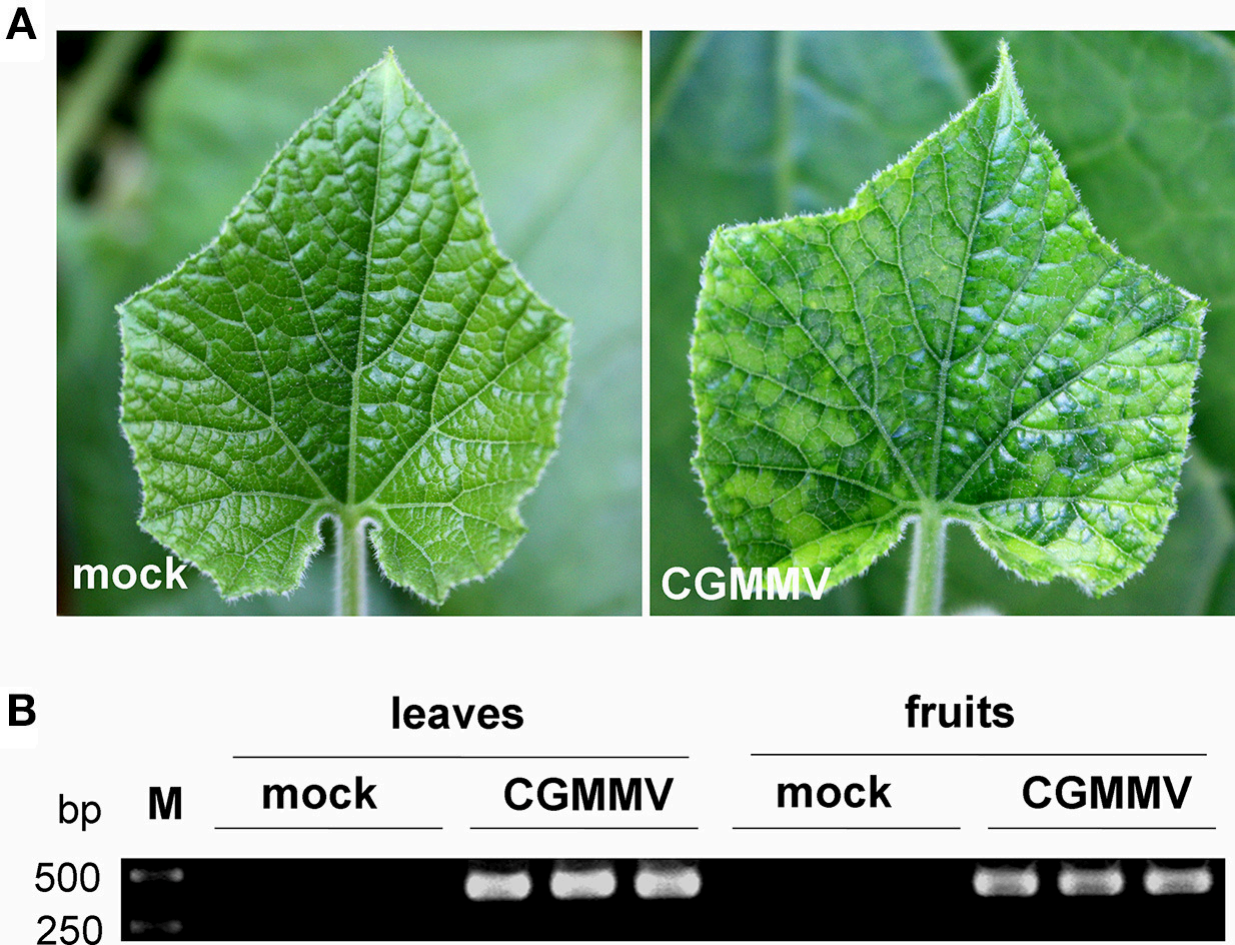
**Symptoms of CGMMV on leaves of ***L. siceraria*** and detection of CGMMV in leaves and fruits of ***L. siceraria*** through RT-PCR. (A)** the typical green mottle mosaic symptom on CGMMV-infected leaves 14 days after inoculation (right panel), but not on mock leaves (left panel). **(B)** RT-PCR detection of CGMMV in CGMMV-infected and mock leaves and fruits of *L. siceraria* (three replicates). Clear bands were observed (confirmed by sequencing) in both leaves and fruits of CGMMV-infected samples, whereas no bands were detected in mock samples.

Cucumber green mottle mosaic virus (CGMMV)-infected leaves of bottle gourd showed the typical green mottle mosaic symptom 14 days after inoculation (Figure 1A), while the infected fruits had only a slight green mottle on the skin. Leaves and fruits were collected from three replicate virus-infected plants and infection with CGMMV was confirmed in each by RT-PCR (Figure 1B). Leaves and fruits from three mock plants were also collected as controls. Small RNAs isolated from extracted total RNAs of these tissues were then used for Illumina high-throughput sequencing.

After the removal of the junk, adapter and repeat reads, total numbers of small RNAs 18–30 nt long obtained from the three virus-infected fruits were 8,558,357 (6,160,591 unique), 9,240,205 (6,799,565 unique) and 5,654,646 (4,129,482 unique). Corresponding numbers from the mock fruits were 9,442,756 (6,399,113 unique), 9,432,345 (6,614,229 unique) and 9,268,640 (6,335,102 unique). From three virus-infected leaves, totals were 10,250,660 (6,413,925 unique), 7,513,459 (4,780,352 unique) and 12,265,181 (7,429,239 unique) and from three mock leaves, there were 8,766,823 (5,268,422 unique), 4,064,336 (2,720,910 unique) and 6,644,494 (3,837,794 unique). An overview of the deep sequencing results is presented in Table 1. In addition, different types of non-coding sRNAs including tRNAs, rRNAs, snoRNAs, and snRNAs were identified while mapping to the Rfam database (Version 12.0) (Figure S1). Interestingly, the numbers of these non-coding sRNAs reads in CGMMV-infected leaves were much larger than in mock leaves, whereas no such pattern was observed for fruits (Figure S1).

**Table 1:** **Summary of deep sequencing results of small RNA libraries from virus-infected and healthy ***L. siceraria*****.

**Category**	**Fruits**						**Leaves**					
	**Replicate 1**		**Replicate 2**		**Replicate 3**		**Replicate 1**		**Replicate 2**		**Replicate 3**	
	**Mock (%)**	**Infected (%)**	**Mock (%)**	**Infected (%)**	**Mock (%)**	**Infected (%)**	**Mock (%)**	**Infected (%)**	**Mock (%)**	**Infected (%)**	**Mock (%)**	**Infected (%)**
Junk reads (unique)	38,257 (0.52)	41,406 (0.58)	40,243 (0.50)	49,056 (0.61)	24,652 (0.34)	29,063 (0.57)	72,490 (1.25)	115,414 (1.49)	42,723 (1.34)	80,746 (1.37)	50,833 (1.18)	118,790 (1.35)
Junk reads (total)	41,065 (0.33)	48,287 (0.40)	43,306 (0.32)	55,305 (0.42)	26,649 (0.21)	33,359 (0.36)	86,896 (0.89)	144,998 (0.88)	51,772 (0.71)	103,055 (0.82)	61,625 (0.73)	155,882 (0.88)
Adapter and Length filter (unique)	777,629 (10.52)	757,192 (10.56)	1,162,594 (14.51)	935,667 (11.71)	766,948 (10.53)	759,138 (14.98)	375,488 (6.48)	842,828 (10.89)	348,862 (10.98)	686,222 (11.68)	378,319 (8.75)	901,498 (10.24)
Adapter and Length filter (total)	2,322,327 (18.66)	2,217,325 (18.59)	3,190,255 (23.73)	2,823,459 (21.57)	2,505,998 (19.82)	2,872,114 (30.77)	798,597 (8.14)	2,542,072 (15.43)	2,975,305 (40.82)	2,266,513 (18.05)	1,635,724 (19.33)	2,610,433 (14.66)
Rfam (unique)	46,796 (0.63)	35,433 (0.49)	48,433 (0.60)	35,086 (0.44)	38,572 (0.53)	32,089 (0.63)	18,787 (0.32)	55,100 (0.71)	21,552 (0.68)	54,019 (0.92)	14,789 (0.34)	47,635 (0.54)
Rfam (total)	424,682 (3.41)	433,138 (3.63)	550,979 (4.10)	413,185 (3.16)	661,347 (5.23)	405,919 (4.35)	73,128 (0.75)	487,950 (2.96)	104,472 (1.43)	418,871 (3.34)	51,803 (0.61)	250,177 (1.41)
Repeats (unique)	3932 (0.05)	3665 (0.05)	3991 (0.05)	3765 (0.05)	3416 (0.05)	2797 (0.06)	1175 (0.02)	5607 (0.07)	1063 (0.03)	4931 (0.08)	1107 (0.03)	5929 (0.07)
Repeats (total)	17,930 (0.14)	8759 (0.07)	12,199 (0.09)	7660 (0.06)	11,231 (0.09)	5984 (0.06)	1971 (0.02)	34,455 (0.21)	1568 (0.02)	25,181 (0.20)	1514 (0.02)	13,177 (0.07)
Clean reads (unique)	6,399,113 (86.55)	6,160,591 (85.93)	6,614,229 (82.58)	6,799,565 (85.12)	6,335,102 (86.98)	4,129,482 (81.48)	5,268,422 (90.98)	6,413,925 (82.85)	2,720,910 (85.64)	4,780,352 (81.37)	3,837,794 (88.76)	7,429,239 (84.36)
Clean reads (total)	9,442,756 (75.87)	8,558,357 (71.74)	9,432,345 (70.17)	9,240,205 (70.60)	9,268,640 (73.31)	5,654,646 (60.59)	8,766,823 (89.32)	10,250,660 (62.22)	4,064,336 (55.76)	7,513,459 (59.85)	6,644,494 (78.51)	12,265,181 (68.90)

**Figure 2:**
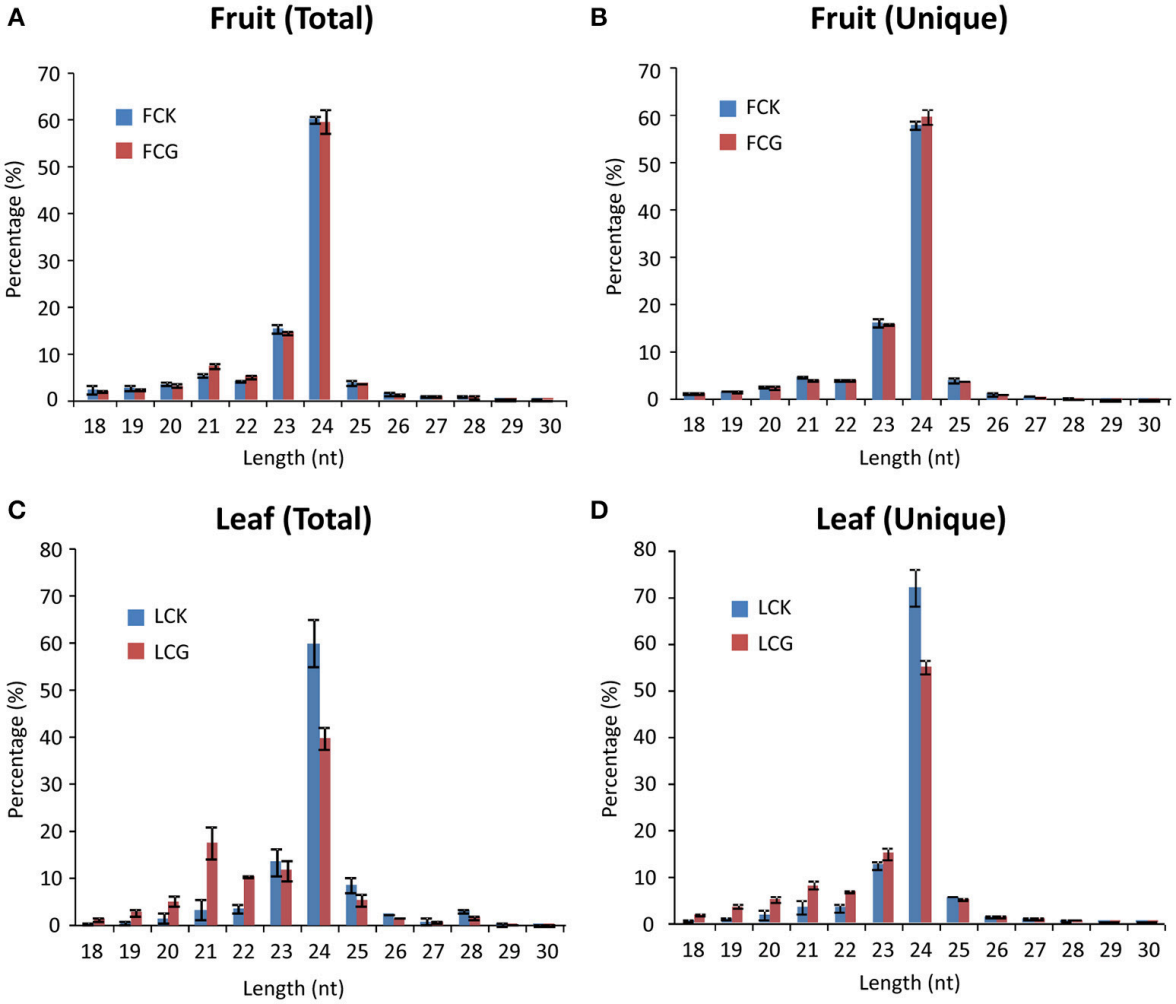
**Size distribution of sRNAs from healthy and CGMMV-infected ***L. siceraria***. (A)** Total sRNAs from fruit. **(B)** Unique sRNAs from fruit. **(C)** Total sRNAs from leaves. **(D)** Unique sRNAs from leaves. FCK, Healthy fruit; FCG, CGMMV Infected fruit; LCK, Healthy leaf; LCG, CGMMV Infected leaf. Error bars indicate ± SD calculated from three biological replicates. The numbers in the horizontal axis indicate length of vsiRNAs, and numbers in the vertical axis indicate percentage of vsiRNAs in healthy or CGMMV-infected samples.

The size distribution of these 12 small RNA libraries was similar. Reads with 24-nt length accounted for most (60–70%) of the total sRNAs, followed by 23-nt (Figure 2). Notably, the percentage of 21- and 22-nt reads in virus-infected leaf samples were significantly larger than in the mock, whereas 24-nt reads were obviously fewer, similar to previous reports (Xia et al., 2014; Li et al., 2016). However, no significant differences of length distribution were observed between mock and infected fruits (Figures 2A,B).

### More vsiRNAs are produced in leaves than in fruits

**Figure 3:**
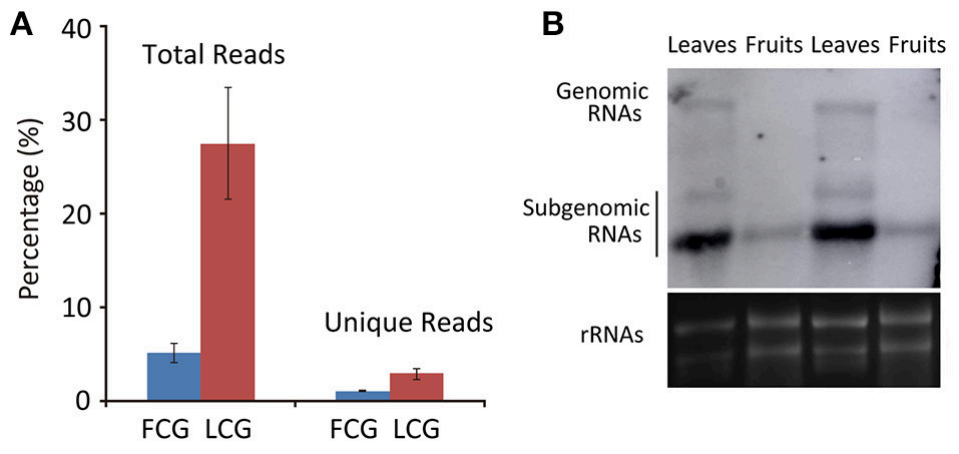
**Abundance of CGMMV derived siRNAs from infected ***L. siceraria***. (A)** Percentage of vsiRNAs in fruit and leaves of *L. siceraria* infected with CGMMV. Error bars indicate ± SD calculated from three biological replicates. FCG, CGMMV Infected fruit; LCG, CGMMV Infected leaf; **(B)** Northern blot detection of CGMMV RNA accumulation in fruit and leaf. Accumulation of CGMMV is much more in leaves compare to fruits for both genomic and subgenomic RNAs. rRNAs were used as control.

To identify CGMMV-derived siRNAs in infected plants, the clean sRNA libraries were mapped to the virus reference genome. The total vsiRNAs accounted for 4.48–6.26% (5.11 ± 0.99%) of the total sRNAs of virus-infected fruits, while the corresponding figures for unique vsiRNAs were 0.95–1.15% (1.06 ± 0.10%). These values are much lower than those from infected leaves where total vsiRNAs accounted for 20.59–30.99% (27.49 ± 5.98%) of the total sRNAs and the corresponding figures for unique vsiRNAs were 2.24–3.47% (2.89 ± 0.62%) (Table 2, Figure 3), indicating that many more vsiRNAs were produced in leaves than fruits. An earlier study of *N. benthamiana* plants infected with Beet necrotic yellow vein virus (BNYVV) showed that the antiviral response was more effective in leaves than in roots; vsiRNAs accumulated more in leaves than in roots, whereas BNYVV mRNA levels were lower in leaves than in roots (Andika et al., 2005). We therefore compared the CGMMV RNA abundance in fruits and leaves using northern blot. Interestingly, higher levels of both genomic RNA and particularly subgenomic RNAs (sgRNAs) accumulated in leaves than in fruits (Figure 3B), correlating positively with the abundance of vsiRNAs (Figure 3A). Our results are consistent with a previous report that increased levels of Rice black-streaked dwarf virus (RBSDV) derived siRNAs in doubly-infected insects (RBSDV and RSV) compared to those infected only with RBSDV was positively correlated with the elevated levels of RBSDV RNA (Li et al., 2013). However, the reasons for these positive or negative relationships between vsiRNAs and mRNA levels in different samples are still not clear.

**Table 2:** Summary of Cucumber green mottle mosaic virus-derived small interfering RNAs (vsiRNAs) from virus-infected L. siceraria^*^.

	**Fruits**			**Leaves**		
	**Replicate 1**	**Replicate 2**	**Replicate 3**	**Replicate 1**	**Replicate 2**	**Replicate 3**
vsiRNAs (unique)	67,105 (1.09%)	64,450 (0.95%)	47,522 (1.15%)	190,382 (2.97%)	165,911 (3.47%)	166,372 (2.24%)
vsiRNAs (total)	535,904 (6.26%)	424,062 (4.59%)	253,195 (4.48%)	3,176,423 (30.99%)	2,321,842 (30.90%)	2,526,004 (20.59%)

* *Small RNA reads from L. siceraria were mapped to the Cucumber green mottle mosaic virus genome with full match and 1 mismatch*.

Since CGMMV can be transmitted by seeds and pollen, it is interesting to investigate whether viruses only in seeds contribute to vsiRNAs production in fruits or viruses in any parts of fruits had such contribution. We hence detected the virus distributions in fruits through tissue immunoblot with antibody of CGMMV. Results showed that viruses were detectable in any parts of virus-infected fruits, indicating the ubiquitous localization of CGMMV in virus-infected fruits (Figure S2). And these results also suggested that, in addition to seeds, viruses in other parts of fruits could also contribute to the production of vsiRNAs in fruits.

### Most vsiRNAs are 21 and 22 nt long

**Figure 4:**
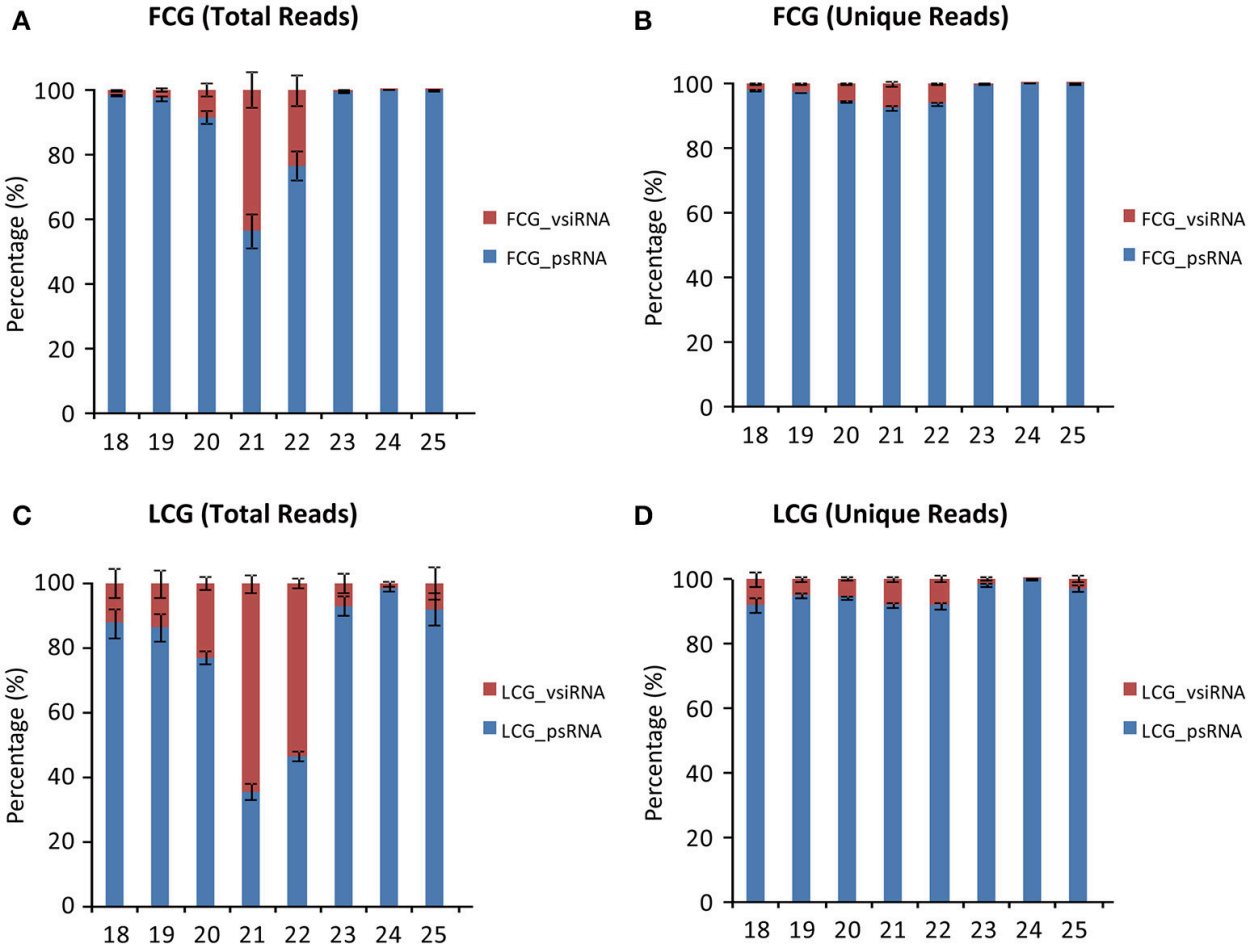
**Percentage of virus derived siRNAs and plant sRNA with different lengths (18–30 nt) from ***L. siceraria*** infected with CGMMV. (A)** Total reads from fruit. **(B)** Unique Reads from fruit. **(C)** Total reads from leaves. **(D)** Unique reads from leaves. FCG_vsiRNA: CGMMV-derived siRNA from infected fruit; FCG_psRNA: Plant sRNA from infected fruit. LCG_vsiRNA: CGMMV-derived siRNA from infected leaf; LCG_psRNA: Plant sRNA from infected leaf. Error bars indicate ± SD calculated from three biological replicates. The numbers in the horizontal axis indicate length of vsiRNAs, and numbers in the vertical axis indicate percentage of vsiRNAs and psRNA in CGMMV-infected samples.

Although 24 nt sRNAs accounted for the largest percentage of total sRNAs, a remarkably high percentage of the 21 and 22 nt sRNAs in infected plants are vsiRNAs, especially in the leaves (64.44 ± 2.62% for 21 nt and 53.54 ± 1.52% for 22 nt) (Figures 4A,C). The increased numbers of 21 and 22 nt sRNAs in infected leaves (as compared to mock-inoculated) may therefore be mainly due to the presence of vsiRNAs (Figures 2C,D). Interestingly, the percentages of unique vsiRNAs, are relatively low (<10%) in both infected leaves and fruits (Figures 4B,D), suggesting that there are very high copy numbers of vsiRNAs in infected plants. The predominance of 21 and 22 nt vsiRNAs has been reported in various eukaryotic organism (Deleris et al., 2006; Donaire et al., 2008; Yan et al., 2010; Li et al., 2013; Mitter et al., 2013; Xia et al., 2014; Yang et al., 2014). This suggests that homologs of DCL4 (production of 21 nt vsiRNA) and DCL2 (production of 22-nt vsiRNA) in *L. siceraria* are actively involved in antiviral defense and play important roles in response to CGMMV infection (Xie et al., 2004; Deleris et al., 2006).

### vsiRNAs are predominantly produced from viral positive strand RNAs in leaves but not in fruits

**Figure 5:**
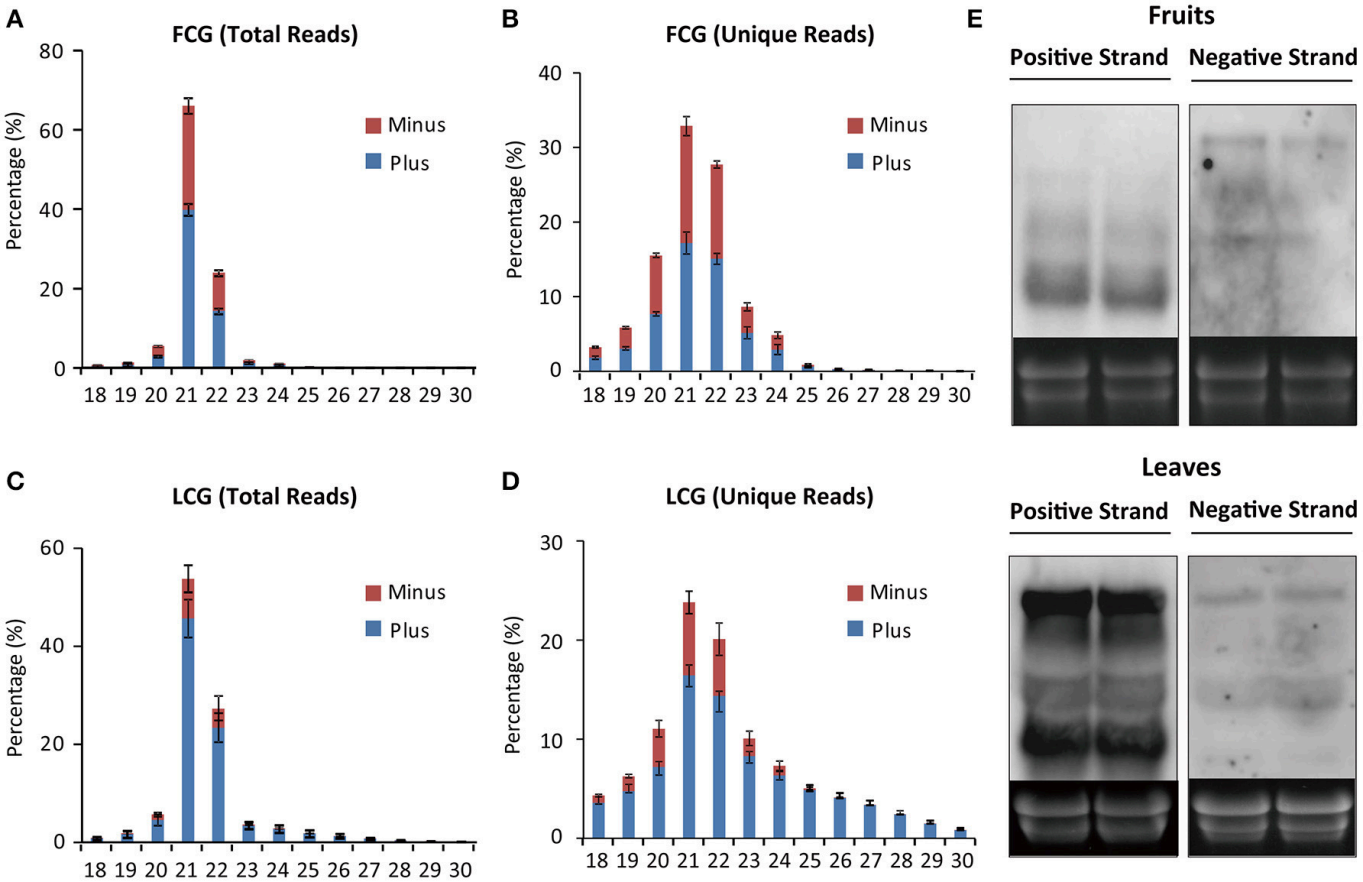
**Size distribution of CGMMV-derived siRNAs from infected ***L. siceraria***. (A)** Total vsiRNAs from fruit. **(B)** Unique vsiRNAs from fruit. **(C)** Total vsiRNAs from leaves. **(D)** Unique vsiRNAs from leaves. FCG, CGMMV Infected fruit; LCG, CGMMV Infected leaf. Error bars indicate ± SD calculated from three biological replicates. The numbers in the horizontal axis indicate length of vsiRNAs, and numbers in the vertical axis indicate vsiRNAs percentage of different lengths in CGMMV-infected samples. **(E)** Northern blot detection of positive and negative-stranded CGMMV RNAs in fruits and leaves infected *L. siceraria*. Two samples were used for each analysis.

The numbers of vsiRNAs derived from positive or negative strand viral RNA were also compared. In infected leaves, many more vsiRNAs were produced from the positive strand viral RNA irrespective of vsiRNA length (Figures 5C,D), which is similar to the results from Cymbidium ringspot virus (CymRSV) and Tobacco rattle virus where vsiRNAs were predominantly from the viral positive strand RNA (Molnár et al., 2005). Furthermore, it has been demonstrated experimentally that secondary structures within the CymRSV single-stranded RNA strands could serve as substrates for DCL-mediated cleavage (Molnár et al., 2005), which might be also one of the reasons for the asymmetry in strand polarity of vsiRNAs in CGMMV infected leaves. Here, we tried to predict the potential secondary structure within the CGMMV positive strand RNA, but no clear relationship was observed between the predicted secondary structure and vsiRNAs with relative high abundance (data not shown). Thus, secondary structure might not the main reason for the asymmetry in strand polarity of vsiRNAs in CGMMV infected leaves. We next detected the accumulation of positive and negative-stranded CGMMV RNAs in leaves to investigate whether this vsiRNA asymmetry polarity was related with the different ratio of positive and negative-stranded CGMMV RNAs in leaves. Results showed that positive-stranded CGMMV RNAs were accumulated much more than negative ones (Figure 5E), which suggests that the vsiRNA asymmetry polarity in leaves might resulted from the high ratio of positive-stranded RNAs to negative ones.

Meanwhile, interestingly, in fruits, vsiRNAs were almost equally from the positive and negative strands of viral RNA (Figures 5A,B). Northern blot showed that the total positive-stranded CGMMV RNAs were accumulated at a similar level to negative ones in fruits of *L. siceraria* according to the size and density of the bands (Figure 5E). However, bands with high density for positive-stranded RNAs were clearly lower compare to negative-stranded ones in blotting (Figure 5E), which probably indicates the complicated composition of CGMMV RNAs with positive-stranded or negative-stranded forms. For a dsRNA virus, almost equal numbers of positive and negative vsiRNAs were generated, suggesting that the dsRNA genome or dsRNA RIs are the target of host Dicer as reported previously (Wu et al., 2010; Li et al., 2013). For ssRNA viruses, approximately equal proportions of positive and negative vsiRNAs have sometimes also been reported where vsiRNAs were mainly derived from viral dsRNA RIs (Aliyari et al., 2008; Wu et al., 2010). This suggests that dsRNA RIs of CGMMV may serve as the major substrates for vsiRNAs production in fruits. Here, we found that the different ratio of positive and negative-stranded CGMMV RNAs in leaves and fruits might be positively correlated to the proportions of positive and negative vsiRNAs.

**Figure 6:**
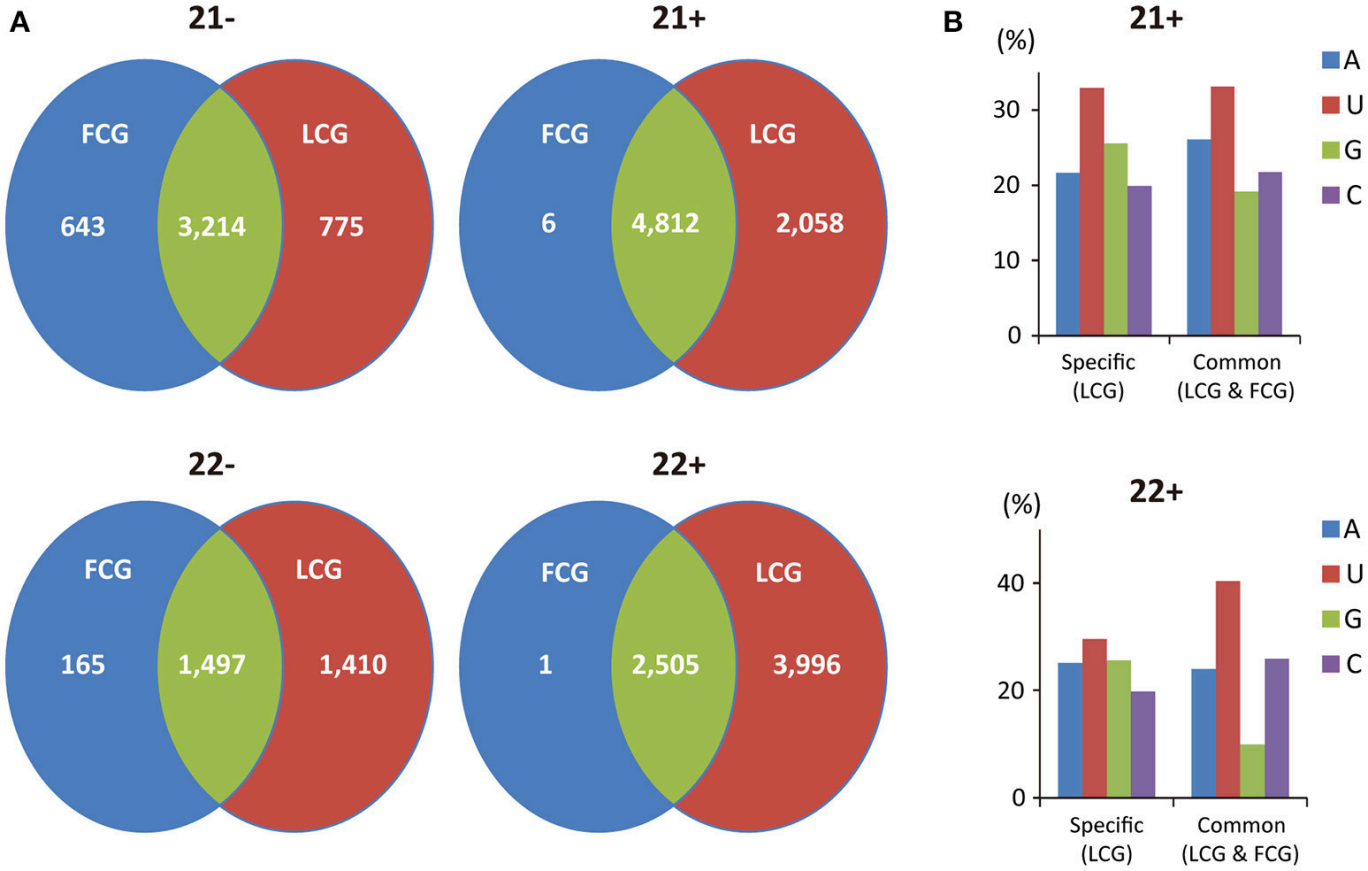
**Profile of tissue-specific and common CGMMV-derived siRNAs in infected ***L. siceraria***. (A)** Abundance of tissue-specific and common vsiRNAs with the lengths of 21 and 22 nt in *L. siceraria*. Much higher numbers of vsiRNAs are specifically produced in leaves compare to fruits, especially for plus strand of vsiRNAs. **(B)** Distribution pattern of the 5′ nt in leaf-specific and common positive vsiRNAs with lengths of 21 and 22 nt. FCG, CGMMV Infected fruit; LCG, CGMMV Infected leaf. 22- or 21- indicate vsiRNAs with length of 22 or 21 nt derived from negative strand of viral genome, and 22+ or 21+ indicate vsiRNAs with length of 22 or 21 nt derived from positive strand of viral genome.

The tissue-specific distribution of vsiRNAs was analyzed further for the 21 and 22 nt vsiRNAs which composed the majority of all vsiRNAs. Only six 21 nt and one 22 nt positive vsiRNAs were produced specifically in fruits while 2058 and 3996 positive vsiRNAs were identified to be specifically produced in leaves for those lengths (Figure 6A), which might be also due to the different ratio of positive and negative-stranded CGMMV RNAs in fruits and leaves of *L. siceraria*.

### vsiRNAs hotspots in fruits, but not in leaves, were only present in the 5′-terminal and 3′-terminal regions of the positive strand

**Figure 7:**
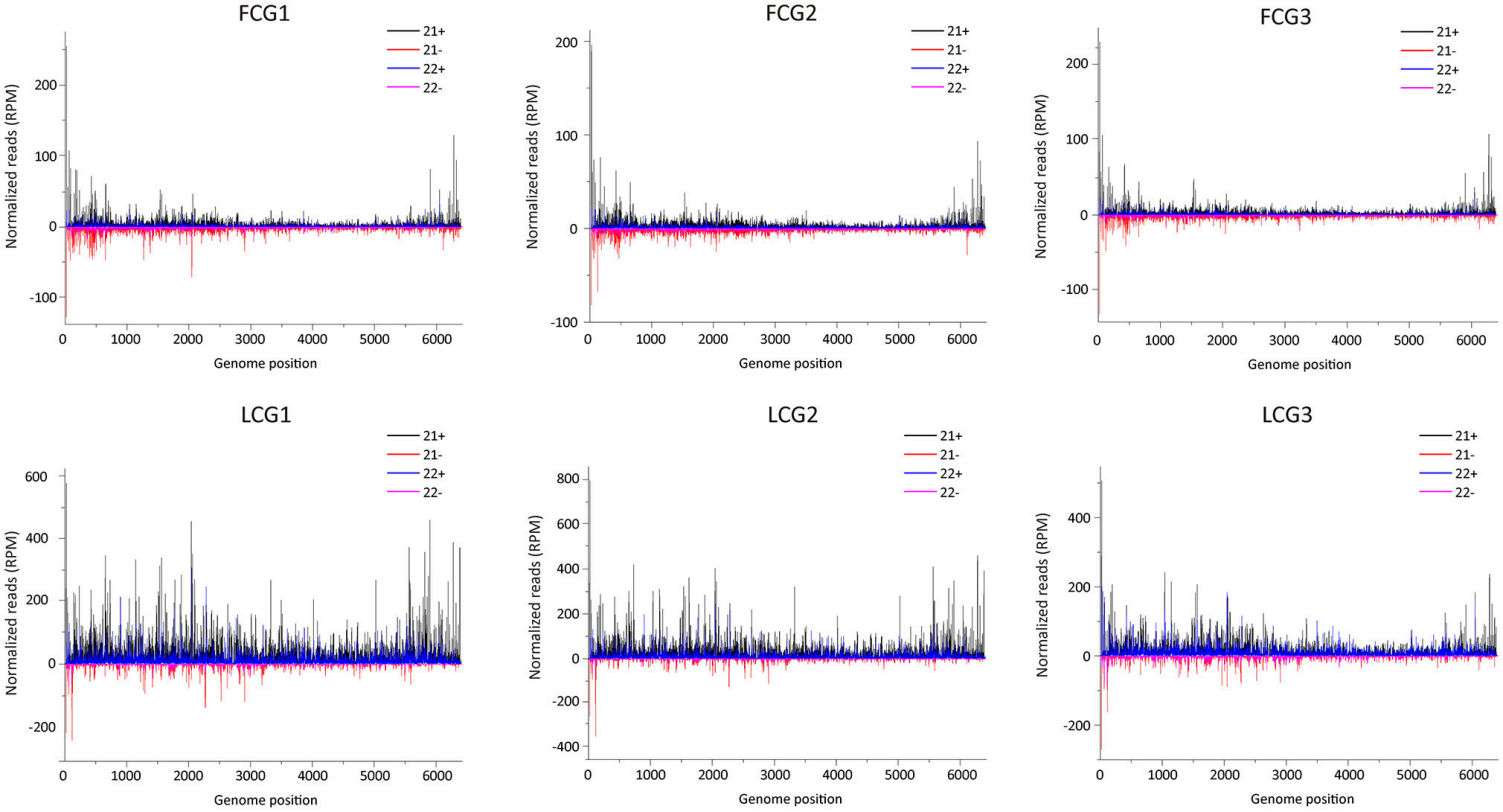
**Distribution of CGMMV-derived siRNAs along the viral genome**. FCG1, FCG2, FCG3: CGMMV Infected fruit (three replicates); LCG1, LCG2, LCG3: CGMMV Infected leaf (three replicates); Color coding indicates viral sRNAs derived, respectively, from the positive (+) and negative genomic strands (−). All reads in this analysis were redundant and normalized.

To examine the distribution pattern of vsiRNAs within the CGMMV genome, 21 and 22 nt long vsiRNAs of all infected libraries were aligned to the virus genome. These vsiRNAs (from both leaves and fruits) cover the entire CGMMV genome (Figure 7), consistent with the previous report (Li et al., 2016). There were strong vsiRNAs preferences to the 5′ terminal of viral negative strand in both fruits and leaves, suggesting that these regions are preferentially cleaved by the host Dicer in fruits (Figure 7). For positive strand, vsiRNAs hotspots were only present in the 5′-terminal and 3′-terminal in fruits, while multiple hotspots were identified for leaves (Figure 7). Recent reports indicated that the production of vsiRNA hotspots in the 3′ region of a virus genome could be ascribed to the presence of viral sgRNAs (Ruiz-Ruiz et al., 2011; Silva et al., 2011; Visser et al., 2014). The CP of CGMMV is expressed from a 3′ terminal sgRNA which might explain the presence of vsiRNA hotspots in the CP region. In addition, we found that many vsiRNAs produced in the 3′ tRNA-like structure region, and the mechanism for this needs further investigation. Previous studies indicated that hairpin structures in single stranded viral genomes can also contribute to the production of vsiRNAs (Molnár et al., 2005; Du et al., 2007). To identify potential secondary structures that might be related to the generation of the vsiRNA hotspots, approximately 300 bp of the CGMMV 5′ and 3′ regions were selected and analyzed. However, no obvious relationship was found between the predicted secondary structures and vsiRNA hotspots region (data not shown). The correlation between vsiRNAs hotpots and secondary structure of the viral genome is still not clear (Donaire et al., 2009). The identification of hotspots for CGMMV derived siRNAs may help select efficient target regions within the genome that can be targeted with artificial siRNA hairpins in future research.

### Different distribution patterns of GC content and 5′-terminal nucleotide of vsiRNAs in leaves and fruits

Previous studies have shown that vsiRNAs are preferentially produced from GC-rich regions and vsiRNAs tend to have a higher GC content than that of the entire viral genome (Ho et al., 2007; Yan et al., 2010). However, the GC content of vsiRNAs (21 and 22 nt) from the positive strand of fruits and leaves was similar to that of the CGMMV genome (Table 3). Interestingly, the GC content of vsiRNAs (21 and 22 nt) from the negative strand was higher in fruits than in leaves (Table 3), indicating a tendency for these negative strand vsiRNAs in leaves to be produced from regions with lower GC content. Furthermore, since vsiRNAs hotspots were commonly identified in the CGMMV 5′ and 3′ regions, 300 bp of these region were also examined. Surprisingly, the 5′-end has GC content of 42.3% which is similar to the GC content of the full genome (43.0%), while the 3′-end has higher GC content (48.3%; Table 3) which might explain the hotspots for vsiRNAs in fruit.

**Table 3:** Nucleotide composition (%) of the unique CGMMV-derived small RNAs (with lengths of 21 and 22 nt) and of the CGMMV genomic sequence^*^.

	**−GC(%)**	**−A(%)**	**−U(%)**	**−G(%)**	**−C(%)**	**GC(%)**	**A(%)**	**U(%)**	**G(%)**	**C(%)**
Fruit 21 nt	42.83 ± 0.35	31.13 ± 0.30	26.04 ± 0.13	18.91 ± 0.16	23.92 ± 0.20	43.76 ± 0.25	25.54 ± 0.19	30.70 ± 0.07	23.84 ± 0.09	19.92 ± 0.16
Fruit 22 nt	42.07 ± 0.41	31.39 ± 0.31	26.54 ± 0.27	18.35 ± 0.18	23.73 ± 0.30	43.06 ± 0.44	25.85 ± 0.25	31.09 ± 0.19	23.38 ± 0.20	19.68 ± 0.25
Leaf 21 nt	39.33 ± 0.82	31.76 ± 0.19	28.91 ± 0.63	17.15 ± 0.47	22.18 ± 0.38	43.28 ± 0.20	25.43 ± 0.26	31.29 ± 0.46	23.26 ± 0.30	20.02 ± 0.10
Leaf 22 nt	38.54 ± 1.02	31.94 ± 0.27	29.52 ± 0.82	16.71 ± 0.63	21.83 ± 0.41	42.64 ± 0.17	25.73 ± 0.18	31.64 ± 0.33	22.91 ± 0.23	19.73 ± 0.08
CGMMV 5′-300 bp	−	−	−	−	−	42.3	34.7	23.0	19.7	22.7
CGMMV 3′-300 bp	−	−	−	−	−	48.3	20.7	31.0	26.7	21.7
CGMMV	−	−	−	−	−	42.99	25.69	31.32	23.82	19.17

* *Small RNA reads from L. siceraria were mapped to the CGMMV genome with full match and 1 mismatch*.

The 5′ terminal nucleotide of small RNAs is important for the sorting of small RNAs into AGO complexes in plants (Mi et al., 2008; Takeda et al., 2008). Our results indicated that 5′ terminal nucleotide of vsiRNAs (21 and 22 nt) from the negative strand was mostly frequently A in fruits or U in leaves, while for the positive strand, the nucleotide was mostly U in both fruits and leaves (Table 4). A 5′ terminal G is underrepresented in both leaves and fruits irrespective of polarity (Table 4). A U preference for the 5′ terminal nucleotide has also been demonstrated in other plants (Donaire et al., 2009; Qi et al., 2009). In *Arabidopsis*, AGO2 and AGO4 preferentially recruit small RNA with a 5′ terminal A, while AGO1 harbors miRNAs with a 5′ terminal of U (Mi et al., 2008). Our data suggest that both AGO2 and AGO4 actively recruit vsiRNAs in leaves and fruits, while AGO1 tends to be involved in the recruitment of negative strand vsiRNAs in fruits. The different 5′ terminal nucleotide preference of vsiRNA (negative strand) for A in fruits and U in leaves suggests that multiple AGO complexes might be involved in varying degrees during anti-viral defense in different tissues.

**Table 4:** First Nucleotide (%) of the unique CGMMV-derived small RNAs (21 and 22 nt)^*^.

	**−A(%)**	**−U(%)**	**−G(%)**	**−C(%)**	**A(%)**	**U(%)**	**G(%)**	**C(%)**
Fruit 21 nt	30.32 ± 0.38	26.66 ± 0.70	16.19 ± 0.80	26.83 ± 0.46	25.04 ± 0.28	31.20 ± 0.63	20.84 ± 0.68	22.92 ± 0.56
Fruit 22 nt	30.05 ± 0.51	26.87 ± 1.39	13.09 ± 0.69	30.00 ± 1.25	25.19 ± 0.47	31.27 ± 1.64	17.15 ± 0.73	26.38 ± 1.59
Leaf 21 nt	28.75 ± 1.28	31.82 ± 2.90	11.51 ± 2.04	27.92 ± 0.58	23.04 ± 1.26	35.79 ± 2.71	15.34 ± 2.09	25.82 ± 0.65
Leaf 22 nt	27.28 ± 1.81	32.45 ± 2.70	9.62 ± 1.97	30.65 ± 1.03	22.98 ± 1.40	34.24 ± 1.69	14.41 ± 1.14	28.36 ± 0.85

* *Small RNA reads from L. siceraria were mapping to CGMMV genome with full match and 1 mismatch*.

Finally, we compared the distribution patterns of the 5′ nt between leaf-specific and common positive vsiRNAs with lengths of 21 and 22 nt. Leaf-specific positive vsiRNAs 21 nt in length had an increased percentage of G at the 5′ compared with common vsiRNAs, while the percentage of A was decreased (Figure 6B). For 22 nt vsiRNAs, the percentage of G was also increased but at the expense of C and U (Figure 6B). The different distributions of the 5′ nt for leaf-specific and common vsiRNAs may suggest the irreplaceable roles of leaf-specific vsiRNAs in antiviral defense.

## Conclusion

In this study, NGS sequencing of sRNAs was performed to investigate profiles of CGMMV-derived siRNAs in infected leaves and fruits of *L. siceraria*. Different vsiRNA patterns of abundance, polarity, hotspot distribution, GC content and 5′-terminal nucleotide were observed in infected leaves and fruits. Furthermore, infected leaves have large numbers of leaf-specific vsiRNAs with a distinct 5′ nt. To our knowledge, this provides the first high-resolution comparison of vsiRNA profiles between different tissues of the same host plant.

## Author contributions

JC and FY conceived and designed the experiments. JL, HZ, CZ, KH, JP, and YL performed the experiments on viruses. JZ, PX, XW, and GL performed the experiments on plants. JL, HZ, JC, and FY analyzed data and wrote the manuscript.

## Funding

This work was financially supported by the Special Fund for Agro-scientific Research in the Public Interest (201303028, 201403032), the International Science & Technology Cooperation Program of China (2015DFA30700) and Fund for Research in the Public Interest of Zhejiang Province (2015C32042).

### Conflict of interest statement

The authors declare that the research was conducted in the absence of any commercial or financial relationships that could be construed as a potential conflict of interest.

## Supplementary material

Figure S1 **Categories of non-coding sRNA in small RNA libraries of healthy and CGMMV-infected *L. siceraria***. FCK1, FCK2, FCK3: Healthy fruit (three replicates); FCG1, FCG2, FCG3: CGMMV Infected fruit (three replicates); LCK1, LCK2, LCK3: Healthy leaf (three replicates); LCG1, LCG2, LCG3: CGMMV Infected leaf (three replicates).

Figure S2 **Tissue immunoblot analysis for detection of CGMMV in virus-infected fruit**. The transaction of fresh virus-free (MOCK) and virus-infected and fruits were shown in upper panels, while the corresponding tissue immunoblot with antibody of CGMMV were shown in bottom panels, which revealed the obvious blue signals in any parts of virus-infected fruits, indicating the ubiquitous localization of CGMMV in virus-infected fruits.
