# Supplementary figures and images for: Different Virus-Derived siRNAs Profiles between Leaves and Fruits in Cucumber Green Mottle Mosaic Virus-Infected Lagenaria siceraria Plants
Source: Front Microbiol. 2016 Nov 9;7:1797. doi: 10.3389/fmicb.2016.01797 (PMC5101232; doi:10.3389/fmicb.2016.01797)

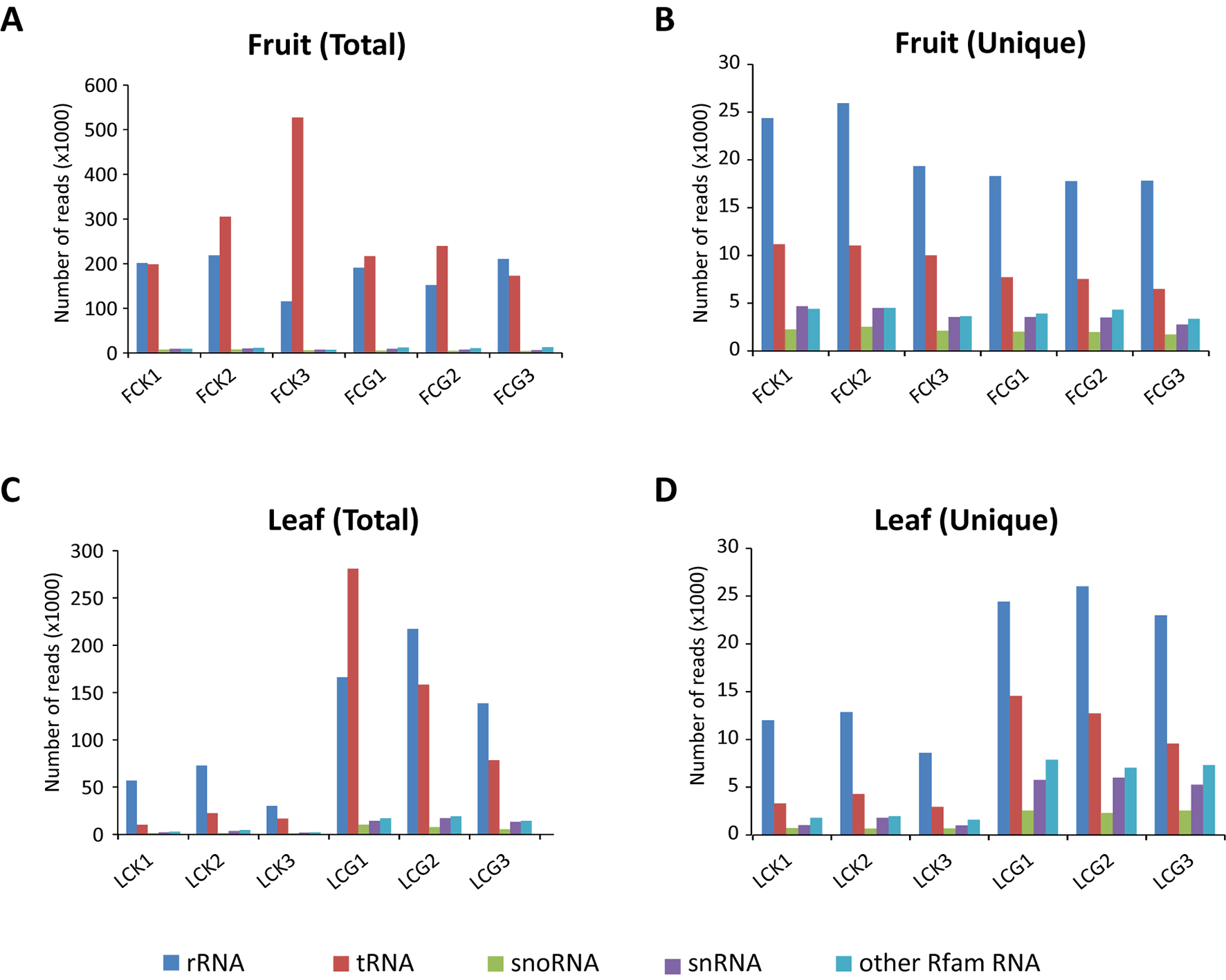

Supplement: Figure S1 — Categories of non-coding sRNA in small RNA libraries of healthy and CGMMV-infected L. siceraria. FCK1, FCK2, FCK3: Healthy fruit (three replicates); FCG1, FCG2, FCG3: CGMMV Infected fruit (three replicates); LCK1, LCK2, LCK3: Healthy leaf (three replicates); LCG1, LCG2, LCG3: CGMMV Infected leaf (three replicates). [file Image1.TIF]

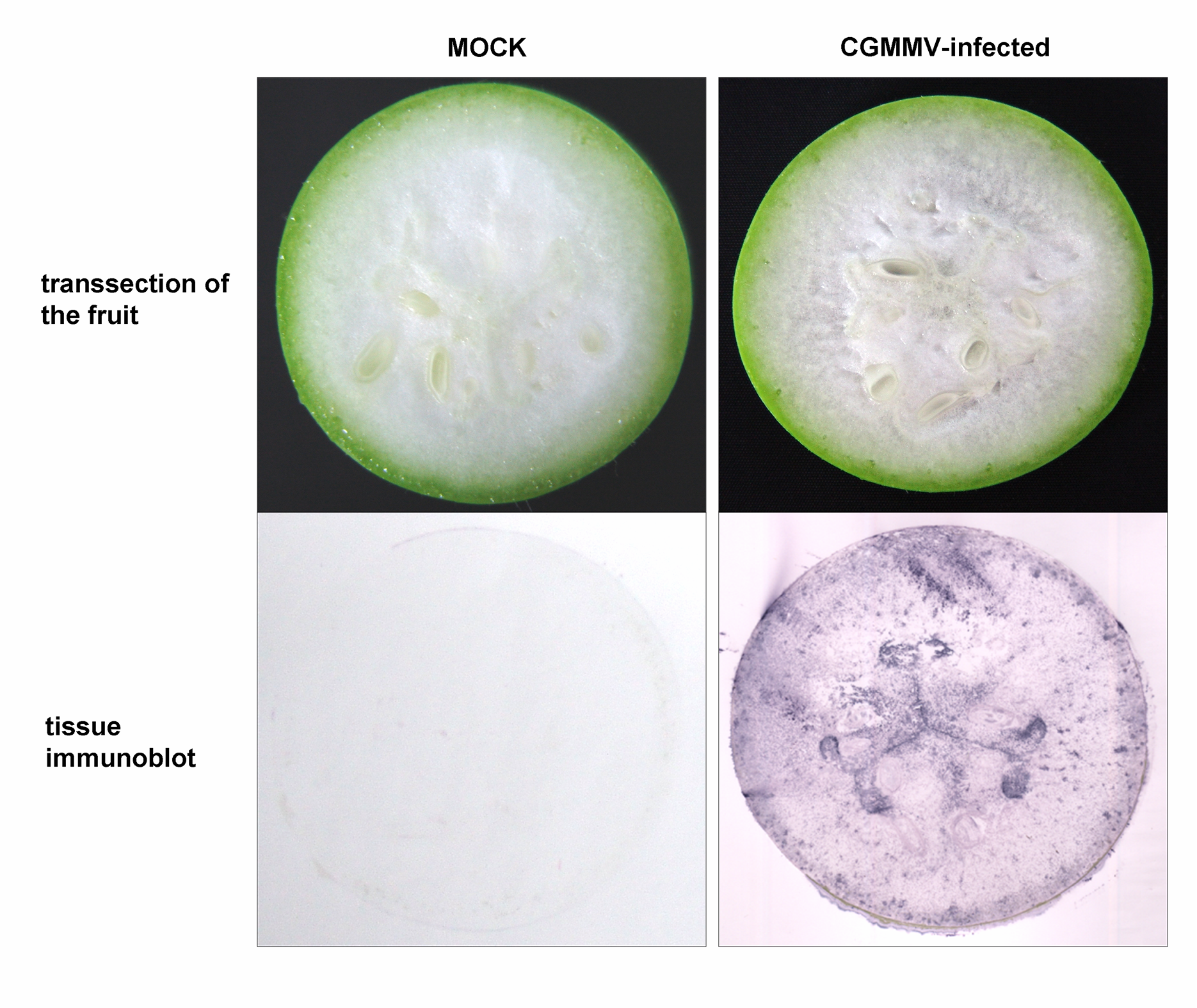

Supplement: Figure S2 — Tissue immunoblot analysis for detection of CGMMV in virus-infected fruit. The transaction of fresh virus-free (MOCK) and virus-infected and fruits were shown in upper panels, while the corresponding tissue immunoblot with antibody of CGMMV were shown in bottom panels, which revealed the obvious blue signals in any parts of virus-infected fruits, indicating the ubiquitous localization of CGMMV in virus-infected fruits. [file Image2.TIF]
